# Hirsutanol A, a novel sesquiterpene compound from fungus *Chondrostereum* sp., induces apoptosis and inhibits tumor growth through mitochondrial-independent ROS production: Hirsutanol A inhibits tumor growth through ROS production

**DOI:** 10.1186/1479-5876-11-32

**Published:** 2013-02-08

**Authors:** Fen Yang, Wen-Dan Chen, Rong Deng, Hui Zhang, Jun Tang, Ke-Wei Wu, Dan-Dan Li, Gong-Kan Feng, Wen-Jian Lan, Hou-Jin Li, Xiao-Feng Zhu

**Affiliations:** 1 State Key Laboratory of Oncology in South China, Cancer Center, Sun Yat-Sen University, 651 Dongfeng Road East, Guangzhou 510060, China; 2 School of Chemistry and Chemical Engineering, Sun Yat-Sen University, Guangzhou 510275, China; 3 Department of Molecular Pathology, The University of Texas MD Anderson Cancer Center, Houston, TX, 77030, USA; 4 School of Pharmaceutical Sciences, Sun Yat-Sen University, Guangzhou 510006, China

**Keywords:** Hirsutanol A, Apoptosis, JNK, Mitochondria, ROS, Cancer

## Abstract

**Background:**

Hirsutanol A is a novel sesquiterpene compound purified from fungus *Chondrostereum* sp. in *Sarcophyton tortuosum*. Our previous studies had demonstrated that hirsutanol A exhibited potent cytotoxic effect on many kinds of cancer cell lines. In the current study, the antitumor activity of hirsutanol A and its molecular mechanisms were investigated.

**Methods:**

Hirsutanol A induced growth inhibition and apoptotic cell death of human colon cancer SW620 cells and human breast cancer MDA-MB-231cells were determined using MTT assay and flow cytometry assay, respectively. The effect of hirsutanol A on intrinsic ROS level and change in mitochondrial membrane potential (△ψm) of different cell lines were also measured by flow cytometry assay. The function of JNK was compromised by JNK siRNA or JNK inhibitor SP600125. The expression of cytochrome *c*, p-JNK, p-c-Jun after treatment with hirsutanol A were detected by Western blot analysis. Finally, the in vivo anti-tumor effect of hirsutanol A was examined in human cancer cell SW620 xenograft model.

**Results:**

The results showed that hirsutanol A significantly induced apoptosis, mitochondrial-independent increase of Reactive Oxygen Species (ROS) level, change of mitochondrial membrane potential, release of cytochrome *c* in human cancer cells. Preventing increase of ROS level using the potent antioxidant N-acetyl-L-cysteine (NAC) markedly decreased hirsutanol A-induced apoptosis. In addition, JNK signaling pathway was activated by hirsutanol A through elevating ROS level. Blockade of JNK signaling pathway by JNK specific inhibitor SP600125 enhanced apoptosis and hirsutanol A-induced ROS accumulation. Also, hirsutanol A exhibited antitumor activity in human cancer cell SW620 xenograft model.

**Conclusion:**

These data suggested that hirsutanol A inhibited tumor growth through triggering ROS production and apoptosis.

## Background

ROS (Reactive Oxygen Species) is a collective term for oxygen derived species, including superoxide anion radical O_2_^.-^, hydroxyl radicals ^.^OH etc. [1]. The basic level of intracellular ROS is considered to be important to promote cell proliferation and differentiation. However, excessive amounts of ROS can contribute to carcinogenesis and cancer progression [2,3]. Therefore, maintaining ROS homeostasis is crucial for normal cell growth and survival [4]. Compared to normal cells, cancer cells with increasing intrinsic ROS are more vulnerable to damage by further ROS insults induced by exogenous agents [5]. Manipulating ROS levels by redox modulation is one way to selectively kill cancer cells sparing normal cells. Hence, the redox system is considered as a new target for anticancer drugs [6,7].

Apoptosis is a highly regulated and organized cell death process, which controls the development and homeostasis of multicellular organisms [8]. Death receptor signaling pathway and mitochondrial pathway are two major pathways mediating apoptosis triggered by different apoptotic stimuli [9,10]. Alterations in the cellular ROS status have been proven to play an important role in apoptotic cell death [11]. Excessive ROS will attack lipids and proteins of mitochondria membrance, leading to severe and irreversible oxidative damage, dysfunction of mitochondria, and release of cytochrome *c*, which in turn activates the caspase-3 initiating mitochondria / cytochrome *c* –mediated apoptosis [12,13].

C-Jun NH2-terminal kinases (JNKs) are strongly activated by oxidative stress, which can induce apoptosis or regulate cellular ROS level by activating its downstream molecule c-Jun [14]. C-Jun is fisrt phosphorylated by JNK and then translocates to the nucleus for further regulating the transcription of target genes including some pro-apoptotic or antiapoptotic proteins such as Bax and Bcl-2 and some redox proteins such as NOX, SOD [15,16].

Hirsutanol A is a novel sesquiterpene compound purified from fungus *Chondrostereum* sp. in *Sarcophyton tortuosum*[17]. Our previous studies showed that Hirsutanol A exerted potent cytotoxic effect on many kinds of human cancer cell lines [18,19]. In this study, we examined the molecular mechanism of Hirsutanol A-induced apoptosis and its antitumor activity in human cancer cell SW620 xenograft model. We demonstrated that Hirsutanol A could induce apoptosis in SW620 and MDA-MB-231 cells and significantly inhibit tumor growth in vivo. Futhermore, we found that hirsutanol A could elevate intrinsic ROS level, and activate mitochondria/ cytochrome *c* signaliing pathway to trigger apoptosis.

## Methods

### Drugs and reagents

**Figure 1:**
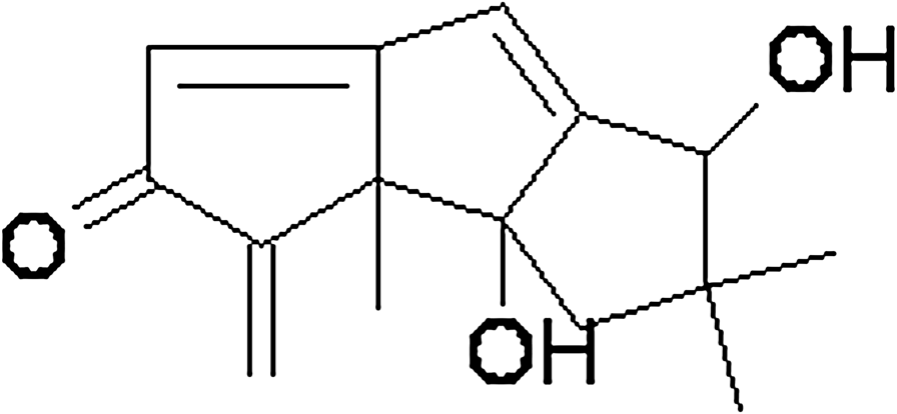
The chemical structure of Hirsutanol A.

Fetal bovine serum and RPMI-1640 media were purchased from Gibco® (New York, USA). 3-(4,5-dime-thylthiazol-2- thiazolyl)-2,5-diphenyltetrazolium bromide (MTT), CM-H2DCF-DA, Dimethyl sulfoxide (DMSO), N-acetyl-L-cysteine (NAC) were obtained from Sigma-Aldrich (St. Louis, USA). 10-Hydroxycamplothecin (HCPT) was purchased from Huangshi Feiyun Pharmaceutical Co., Ltd (Hubei, China). Antibodies against Hsp60, JNK, p-JNK, chemiluminescence reagent were acquired from Cell Signaling Technology (Danvers, MA, USA). Antibodies against GAPDH, Caspase-3, PARP, Cyto-c, p-c-Jun and anti-mouse Ig-G-horseradish peroxidase, anti-rabbit Ig-G-horseradish peroxidase were from Santa Cruz Biotechnology (Santa Cruz, USA). The c-Jun antibody was purchased from Boster Biotech (Wuhan, Hubei, China). Cell lysis was from Upstate Biotech Co (New York, USA). Hirsutanol A, a sesquiterpene compound, was isolated from fungus *Chondrostereum* sp. in *Sarcophyton tortuosum*, and initially dissolved in 100% DMSO at 100nM and stored at −20°C. Its structure is shown in Figure 1.

### Cell lines and cell culture

Human colon cancer cell line SW620 and human breast cancer cell line MDA-MB-231 were cultured in RPMI-1640 media supplemented with 10% heat-inactivated fetal bovine serum, penicillin (50U/ml) and streptomycin (50 μg/ml) at 37°C in 5% (v/v) CO_2_. All experiments were carried out with cells in logarithmic growth phase.

### MTT assay

SW620 and MDA-MB-231 cells were first seeded in 96-well plate at a density of 8,000 cells per well, then treated with different concentrations of hirsutanol A for indicated times or pre-incubated with 1mmol/L antioxidant NAC for 1 h, then cultivated for 72 h at 37°C. 10 μL of 5 mg/mL MTT was added into each well before the termination of experiment. The plates were incubated at 37°C, 5% (v/v) CO_2_ for 4 h. After complete removal of the medium, 100 μL of DMSO was added into each well to dissolve the insoluble purple formazan product. Absorbance values were obtained with a test wavelength of 570 nm. The rates of cell growth inhibition were calculated based on the absorbance values. The 50% inhibitory rates (%) were calculated by the Bliss method: Inhibitory rate = (1-the average OD value of treatment group/ the average OD value of the control group) × 100% [20].

### Annexin V/Propidium Idodide (PI) double-staining assay

Annexin V/PI staining was performed using the Annexin V-fluorescein isothiocyanate apoptosis detection kit. Cells (3.0 × 10^5^ per mL) were seeded into six-well plate with 2 mL in each well, then treated with different concentrations of hirsutanol A for 72 h or pretreated with 1mmol/L NAC or 10 μmol/L SP600125 followed by hirsutanol A for 72 h. Both floating and attached cells were collected, washed with ice-cold PBS twice, then incubated at room temperature in the presence of media binding reagent and Annexin V-FITC for 15min in the dark. After washing with PBS, the cells were re-suspended in ice-cold 1 × binding buffer and treated with 10 μL propidium iodide (30 μg/ml) on ice in the dark. Apoptosis was quantified by flow cytometry (Becton Dickinson) at the wavelength of 488 nm immediately and analyzed by the Cell-Quest software [21].

### Flow cytometry assay and measurement of ROS

Cells were diluted to 3.0 × 10^5^ per mL, seeded into six-well plate with 2 mL in each well. SW620 cells and MDA-MB-231 cells were treated with hirsutanol A for indicated times or treated with various concentrations of hirsutanol A for 24 h, or pre-incubated with 1 mmol/L NAC or 1 μmol/L SP600125 followed by hirsutanol A for 24 h, then incubated with 1 μmol/L (final concentration) CM-H2DCF-DA or DHE florescent dye in dark for 1 h at 37°C. After washing twice with ice cold PBS, cells were centrifuged and re-suspended in PBS. The level of intracellular ROS was detected by flow cytometry with FACS Calibur system and CellQuestPro analysis software [22].

### Immunoblotting analysis

Cells were seeded into six-well plate, treated with hirsutanol A for indicated times or pre-incubated with NAC for 1 h followed by hirsutanol A for 24 h. Cells were harvested and washed twice with PBS and lysed in lysis buffer. The cell lysates were clarified by centrifugation at 12,000 g for 10 min at 4°C and the protein concentration was determined using the Bio-Rad protein assay (Bio-Rad laboratories). SDS-PAGE sample buffer was added to cell lysates. Then the cell lysates were heated at 100°C for 5 min, and cell lysates containing 20–40 μg protein was loaded in each well of 8% (w/v) and 15% (w/v) SDS-PAGE gel. Resolved proteins were electrophoretically transferred to PVDF membrane, which was incubated sequentially with primary antibody and horseradish peroxidase–conjugated second antibody. After washing, the bound antibody complex was detected using an ECL chemiluminescence reagent and XAR film (Kodak) as described by the manufactures [23,24].

### siRNA transfection

The target sequence for JNK-specific siRNA was 5^′^-AAAAAGAAUGUCCUAC CUUCU-3^′^, and control siRNA (no silencing) were synthesized by GenChem Co. One day before transfection, cells were plated in six-well plates with antibiotic-free growth medium at a density of 1.5 × 10^5^ cells per well. When cells grew to a confluency of 30-50% on the second day, transfection was performed by using Opti-MEM media, lipofectamine 2000 and JNK siRNA according to manufacturer’s recommendations. The final concentration of JNK siRNA was 100 nM. After 6 h, the Opti-MEM media was replaced with the antibiotic free growth media and cells were treated with 20 μmol/L hirsutanol A for 3 h.Cells transfected with lipofectamine 2000 were used as control [25].

### Mitochondrial /cytosol fractionation

The isolation of cell mitochondrial and cytosolic fractions was performed using mitochondria/cytosol fractionation kit according to the following protocol. Cells previously treated with 20 μmol/L hirsutanol A for 24 h were harvested at ~850 × g for 2 min and 800 μl of mitochondria isolation reagent A and 10 μl of mitochondria isolation reagent B were added. After 5 min incubation on ice, 800 μl of mitochondria isolation reagent C was added and the mixture was centrifuged at 700 × g for 10 min at 4°C. The supernatant was further centrifuged at 12,000 × g for 15 min at 4°C in order to pellet the crude mitochondria. 500 μl mitochondria isolation reagent C was then added to the pellet before the final centrifugation at 12,000 × g for 5 min at 4°C. The resulting mitochondrial pellet and cytosol fraction were lysed by lysis buffer before further processing [26].

### In vivo antitumor studies

BALB/c nude mice (4 to 6 week-old) were obtained from Guangzhou University of Chinese Medicine. All manipulation was done under sterile conditions. The procedures involving mice and their care were in accordance with National Institutes of Health Guide for the care and use of Laboratory Animals and with the United Kingdom Coordinating Committee on Cancer Research. Tumor xenografts were established by injecting 1 × 10^6^ SW620 cells into the subcutaneous tissue in both flank of nude mice. Mice were randomly divided into six groups and each group contained 6 mice. Treatment was initiated on day 6 after inoculation, by which time the volume of the tumor had reached approximately 50mm^3^. Different concentration of hirsutanol A (20 mg/kg/d, 10 mg/kg/d, 5 mg/kg/d), DMSO, 0.9% (w/v) NaCl Saline (N.S.) and HCPT were administered i.p. for 28 days for the assigned group. Tumor volumes and body weight of the mice were observed. Tumor volumes were calculated by the formula: 0.5 × a × b^2^ in millimeters, where a is the length and b is the width. On day 28 after administration, the mice were sacrificed. The tumor tissues were excised and weighed. Tumor growth inhibition was determined as the ratio of the average tumor weight of the treated group (T) to the average tumor weight of the control group (C) [27].

### Statistical analysis

Data were analyzed by student’s t test with SPSS 11.0 analysis software, and results were considered statistically significant at p < 0.05. Results are presents as mean and standard deviation (±SD).

## Results

### Hirsutanol A inhibited proliferation and induced apoptosis in SW620 and MDA-MB-231 cells

**Figure 2:**
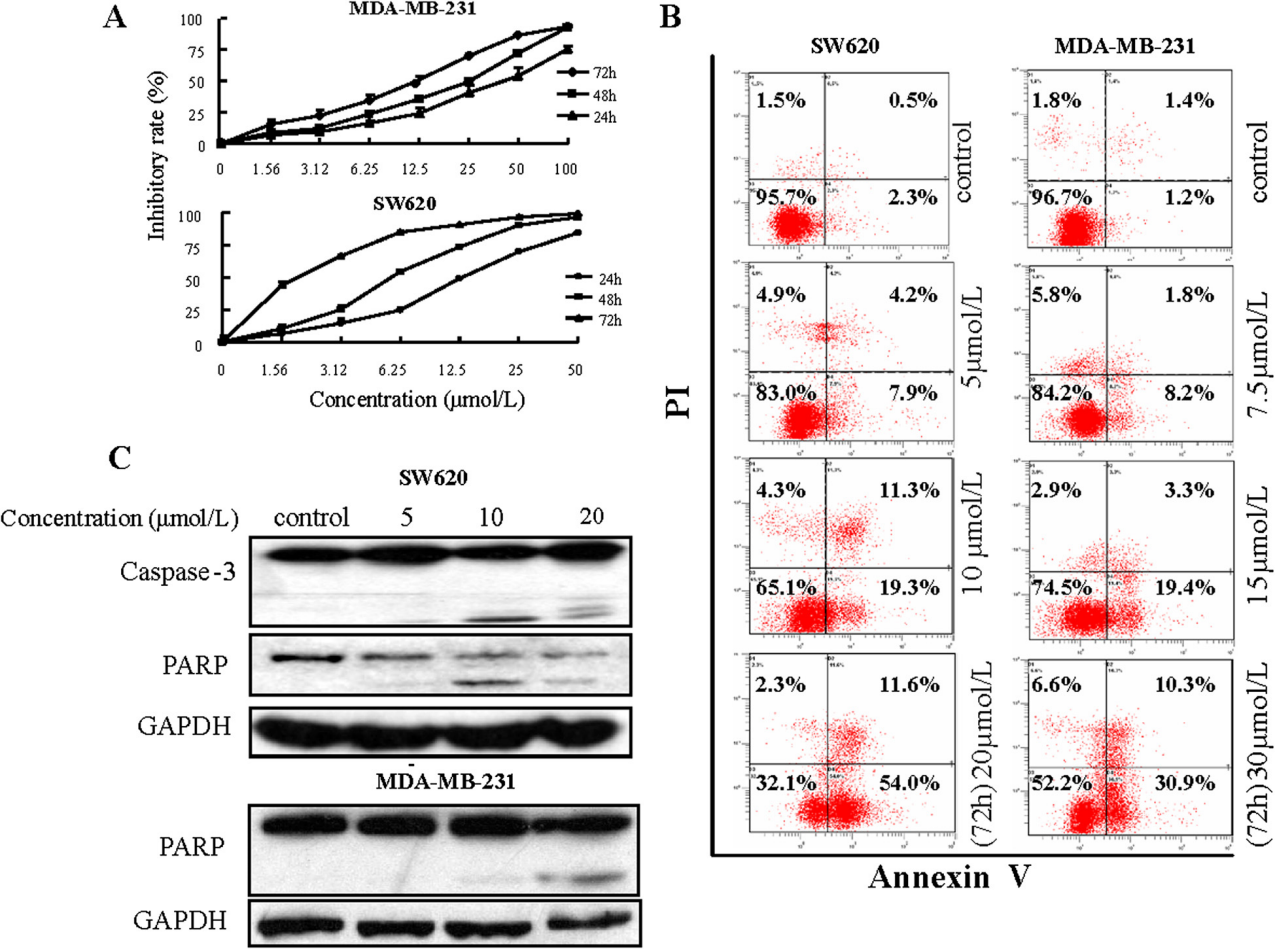
**Growth inhibition and apoptosis induced by hirsutanol A in SW620 and MDA**-**MB**-**231 cancer cells. **Cells were treated with different concentrations of Hirsutnol A for 72 h, 48 h, 24 h respectively. The inhibitory rate was detemined by MTT assay (**A**). Cells were treated with various concentrations of hirsutanol A for 72 h, AnnexinV/PI analysis were used to detect the cell apoptosis (**B**). Proteins were harvested from the SW620 and MDA-MB-231 cells after treatment with indicated concentrations of hirsutanol A for 48 h. Cleavage fraction of PARP and Caspase-3 were detected by immunoblotting assay (**C**). Results are presented as means ± SD from 3 independent experiments.

Using MTT assay, we found that hirsutanol A inhibited cell proliferation in a dose- and time- dependent manner. The half-maximal inhibitory concentration (IC_50_) were 1.90 μmol/L, 6.16 μmol/L, 13.43 μmol/L for SW620 cells and 10.48 μmol/L, 18.01 μmol/L, 35.67 μmol/L for MDA-MB-231 cells after treatment with hirsutanol A for 72, 48, 24 h respectively (Figure 2A). Inhibition of cell growth could be the consequences of the induction of apoptosis, necrosis and cell growth arrest [28]. Thereby, we investigated whether hirsutanol A could induce apoptosis in SW620 and MDA-MB-231 cells. Phosphatidyl serine translocation to the cell surface is an important indicator of early apoptosis [29]. AnnexinV-fluorescein isothiocyanate/propidium iodide staining assay was also employed to monitor the apoptotic cells. The percentages of AnnexinV-positive cells were 2.8%, 12.1%, 45.0%, 65.6% in SW620 and 2.6%, 10.0%, 22.7%, 30.6% in MDA-MB-231 after treatment with various concentrations of hirsutanol A for 72 h. These data indicated that hirsutanol A could induce apoptosis in a dose-dependent manner (Figure 2B). Furthermore, with Western blot analysis we found that pro-caspase-3 was cleaved to form a 17KDa fragment and PARP was cleaved into an 89KD fragment (Figure 2C). These results suggest that hirsutanol A significantly induced apoptosis in SW620 and MDA-MB-231 cells.

### Hirsutanol A induced mitochondrial-independent accumulation of intrinsic ROS

**Figure 3:**
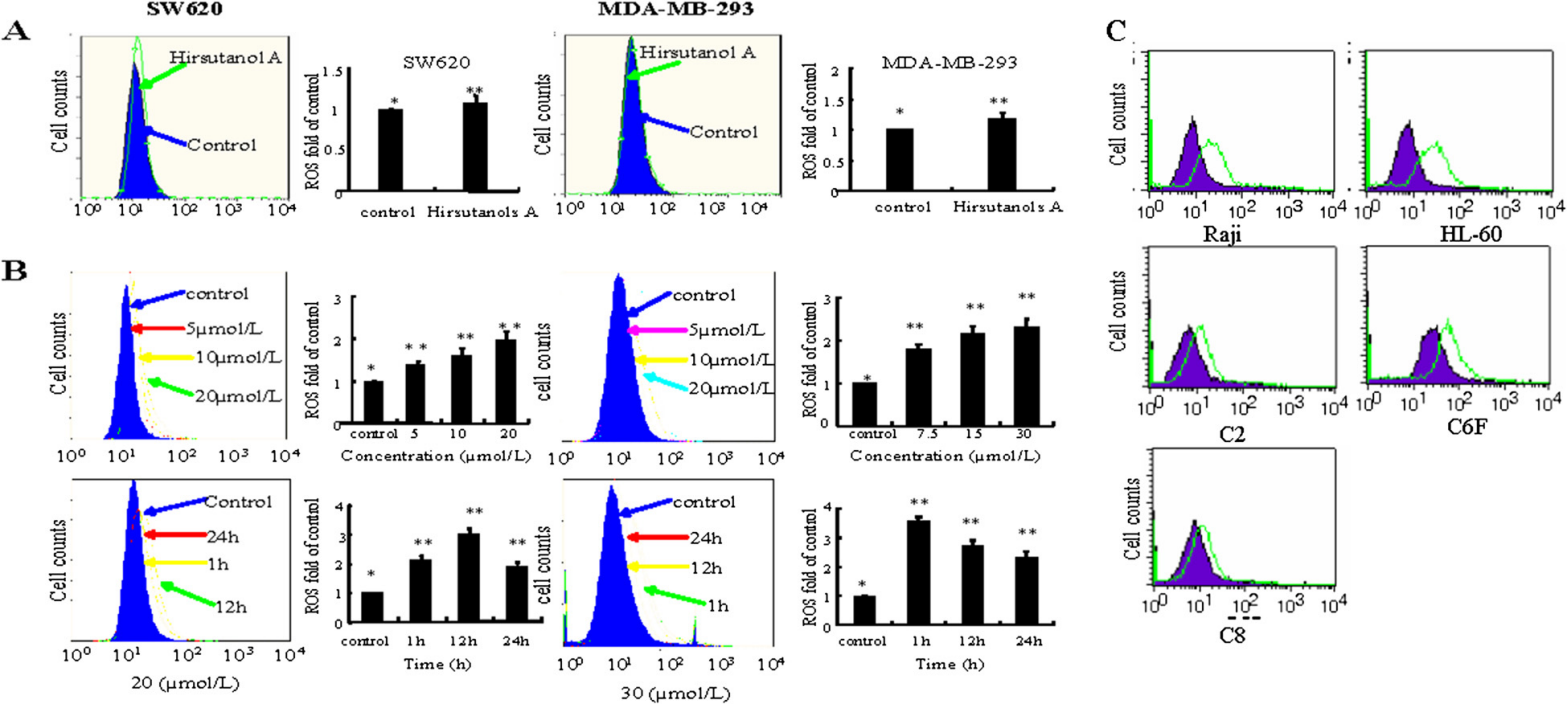
**Hirsutanol A induced mitochondrial**-**independent accumulation of intrinsic ROS in SW620 and MDA**-**MB**-**231 cells. **Cells were stained with DHE for 1 h after exposure to hirsutanol A for 3 h. The cellular O_2_ level was monitored by flow cytometry. Results are presented as means ± SD from 3 independent experiments. (** < 0.05 versus *) (**A**). SW620 and MDA-MB-231 cells were treated with various concentrations of hirsutanol A for 24 h or with 20 μmol/L and 30 μmol/L hirsutanol A for indicated time respectively, and then stained with CM-H2DCF-DA for 1. Results are presented as means ± SD from 3 independent experiments. (** < 0.01 versus *) (**B**). Raji, HL60, C2, C8 and C6F cells were treated with 20 μmol/L hirsutanol A for 5 h, and then stained with CM-H2DCF-DA for 1 hour. The cellular H_2_O_2_ level was monitored by flow cytometry (**C**).

Previous studies had confirmed that hirsutanol A could induce autophagical cell death by causing an accumulation of ROS level in human hepatocellular carcinoma cells [18]. As reactive oxygen species mainly include hydrogen peroxide H_2_O_2_ and superoxide anion radical O_2_^.-^, in the present study, the effect of hirsutanol A on cellular superoxide and hydrogen peroxide level was measured in SW620 cells and MDA-MB-231 cells. The level of superoxide and hydrogen peroxide in cancer cells were analyzed by flow cytometry using DHE and CM-H2DCF-DA as fluorescent probe [30,31]. There was no significant change in DHE fluorescence after treatment with hirsutanol A for 3h but a remarkable increase of CM-H2DCF-DA fluorescence in a dose-dependent fashion (Figure 3A and 3B), suggesting that the ROS induced by hirsutanol A were mainly hydrogen peroxide instead of superoxide. Since accumulation of ROS was mainly caused by the increase of mitochondrial respiratory chain production and decrease of capability for scavenging ROS by the redox system, we thereby investigated whether hirsutanol A-induced increase of ROS was related to mitochondria. C6F cells, a clone of rho-0 cells (mitochondrial respiration defective) derived from HL-60 cells and parental HL-60, were used to detect whether hirsutanol A-induced accumulation of ROS production was mitochondrial respiratory chain related. Results showed that both the parental HL-60 and roh-0 cells (C6F) were highly sensitive to hirsutanol A. C2, C8 cells (rho-0 cells) and parental Raji cells also showed similar effect after treatment with hirsutanol A, which suggested that the accumulation of ROS production were mitochondrial respiratory chain independent (Figure 3C).

### Preventing ROS accumulation by antioxidant agent NAC reduced hirsutanol A-induced apoptosis

**Figure 4:**
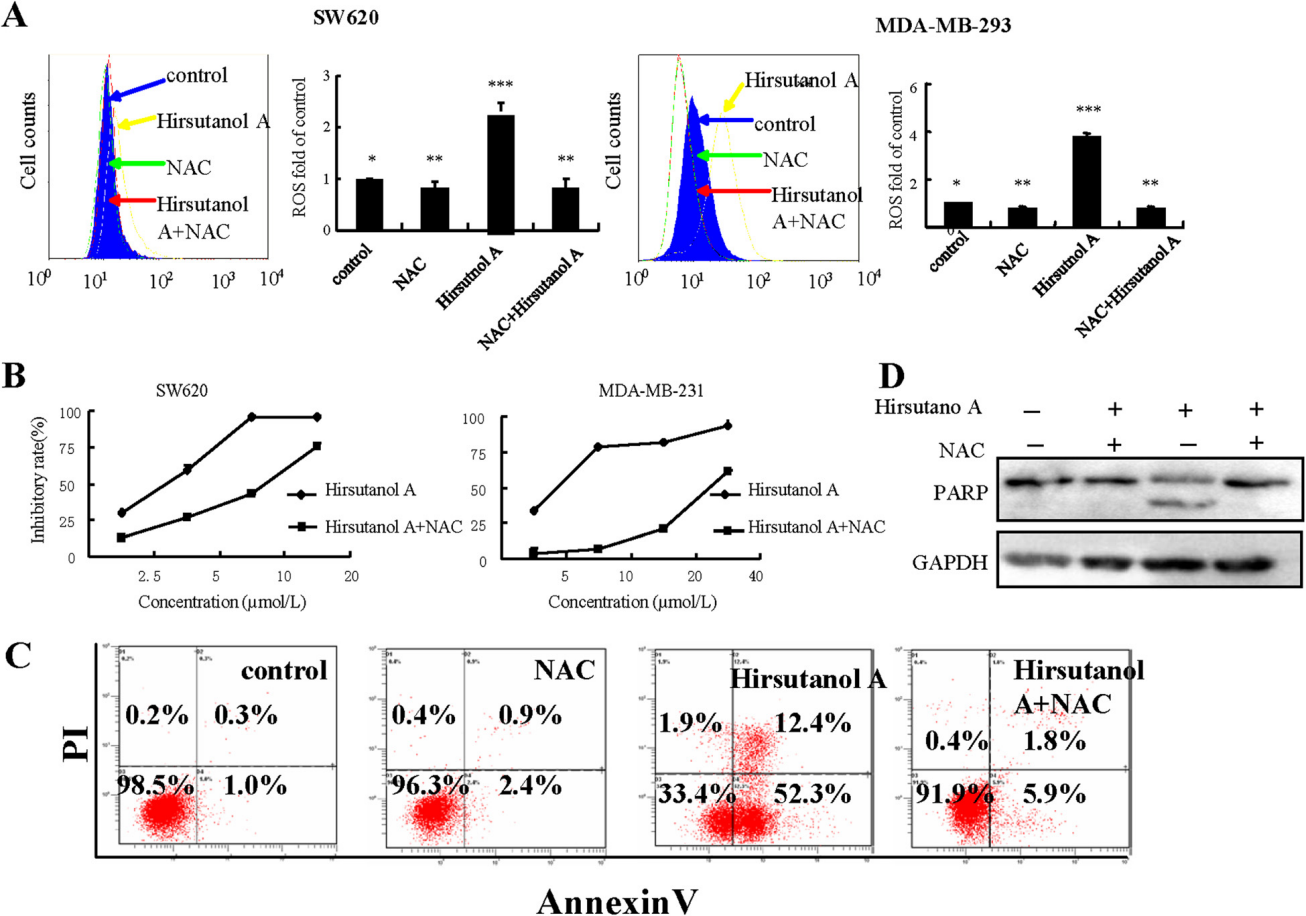
**Preventing ROS accumulation by antioxidant agent NAC reduced hirsutanol A**-**induced apoptosis. **Cells were pre-incubated with NAC for 1h, then treated with hirsutanol A for 3h. The cellular H_2_O_2_ level was monitored by flow cytometry. Results are presented as means ± SD from 3 independent experiments. (** < 0.05 versus *; *** < 0.01 versus *) (**A**). Cells were pre-incubated with 1mmol/L NAC for 1h , then treated with various concentrations of hirsutanol A for 72h. The growth inhibition in SW620 and MDA-MB-231 cells were detected by MTT assay (**B**). Cells were pre-incubated with 1mmol/L NAC for 1h, then treated with various concentrations of hirsutanol A for 48h. Cell apoptosis was detected by AnnexinV/PI analysis (**C**).Cells were pre-incubated with 1mmol/L NAC for 1h, then treated with 20μmol/L hirsutanol A for 24h. The cleavage fraction of PARP was detected by immunoblotting assay (**D**).

Excessive ROS could lead to mitochondrial membrane damage, the release of cytochrome *c* from mitochondrial and cell apoptosis. The evidences of apoptosis and up-regulation of ROS levels in cells treated with hirsutanol A prompted us to investigate whether up-regulation of ROS would resulted in apoptosis. The increase of ROS levels in hirsutanol A-treated cancer cells was prevented by pre-incubation with NAC for 1h. Cell growth inhibition was analyzed using MTT assay and AnnexinV- positive cells were detected by Annexin V/PI double staining assay (Figure 4A to 4C). The results showed that hirsutanol A-induced AnnexinV-positive cells and growth inhibition were significantly reduced. In addition, prevention of ROS accumulation could inhibit the PARP cleavage in hirsutanol A-treated cells (Figure 4D). These data suggested that accumulation of ROS mediated hirsutanol A-induced apoptosis.

### Hirsutanol A activated mitochondria/cytochrome c signaling pathway

**Figure 5:**
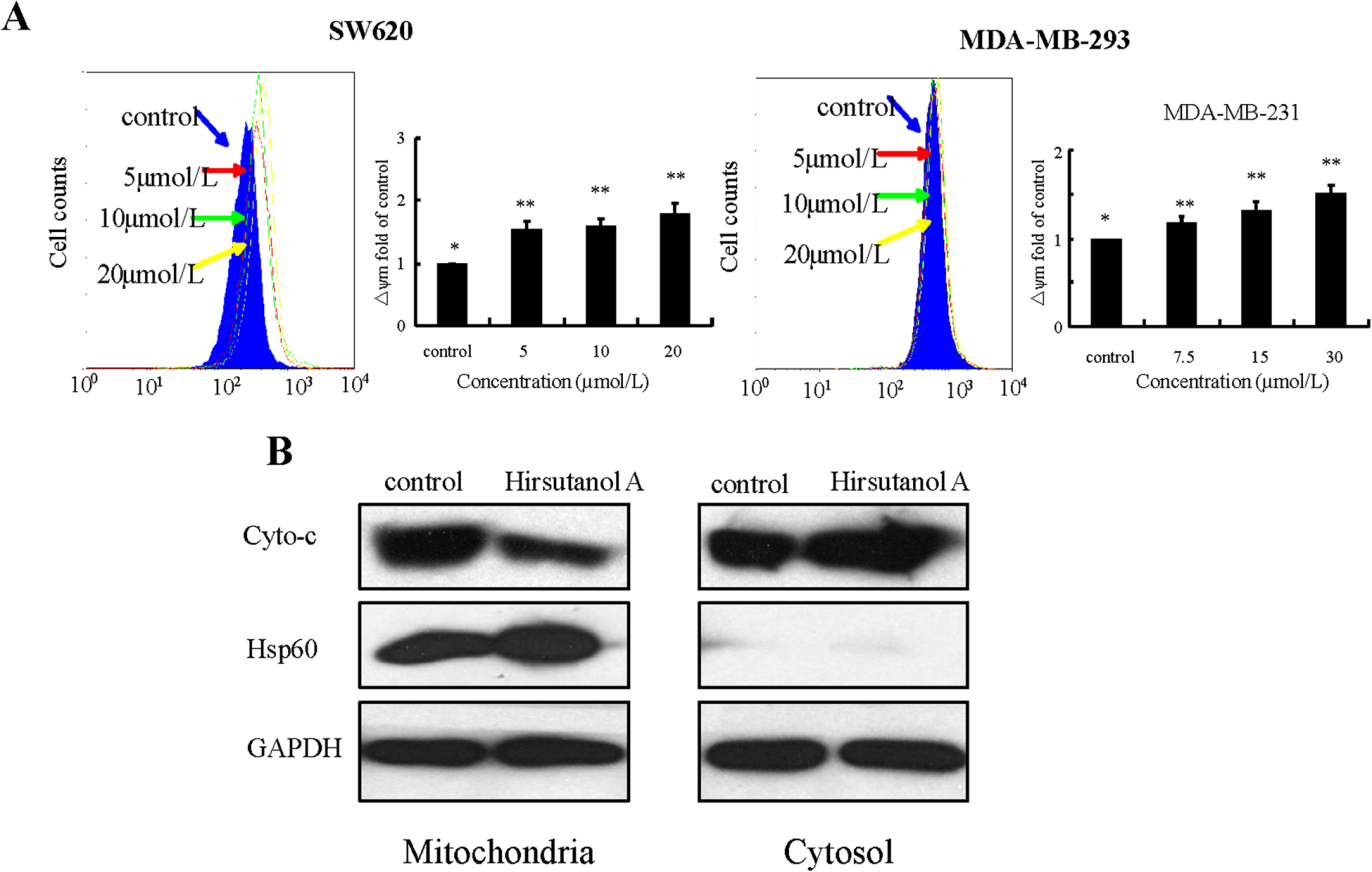
**Hirsutanol A activated mitochondria**/**cytochrome *c *signaling pathway. **Cells were exposed to various concentrations of hirsutanol A for 3h. The △ψm was determined by flow cytometry. Results are presented as means ± SD from 3 independent experiments. (** < 0.01 versus *) (**A**). Cells were first treated with 20μmol/L hirsutanol A for 24h. Proteins obtained from cytosolic and mitochondria fractions were detected by immunoblotting assay (**B**).

To further study whether hirsutanol A induced apoptosis via activation of mitochondria/cytochrome *c* signaling pathway, we examined the change of mitochondrial membrane potential and the release of cytochrome *c* from mitochondria. Mitochondrial membrane potential was elevated after treatment with various concentrations of hirsutanol A (Figure 5A). The expression of cytochrome *c* in mitochondria was down-regulated, whereas cytosolic cytochrome *c* was increased after treatment with hirsutanol A for 24 h (Figure 5B). These data revealed that hirsutanol A induced apoptosis through activation of mitochondria/cytochrome *c* signaling pathway.

### Hirsutanol A activated JNK signaling pathway and blockade of JNK signal pathway increased ROS level and cell apoptosis

**Figure 6:**
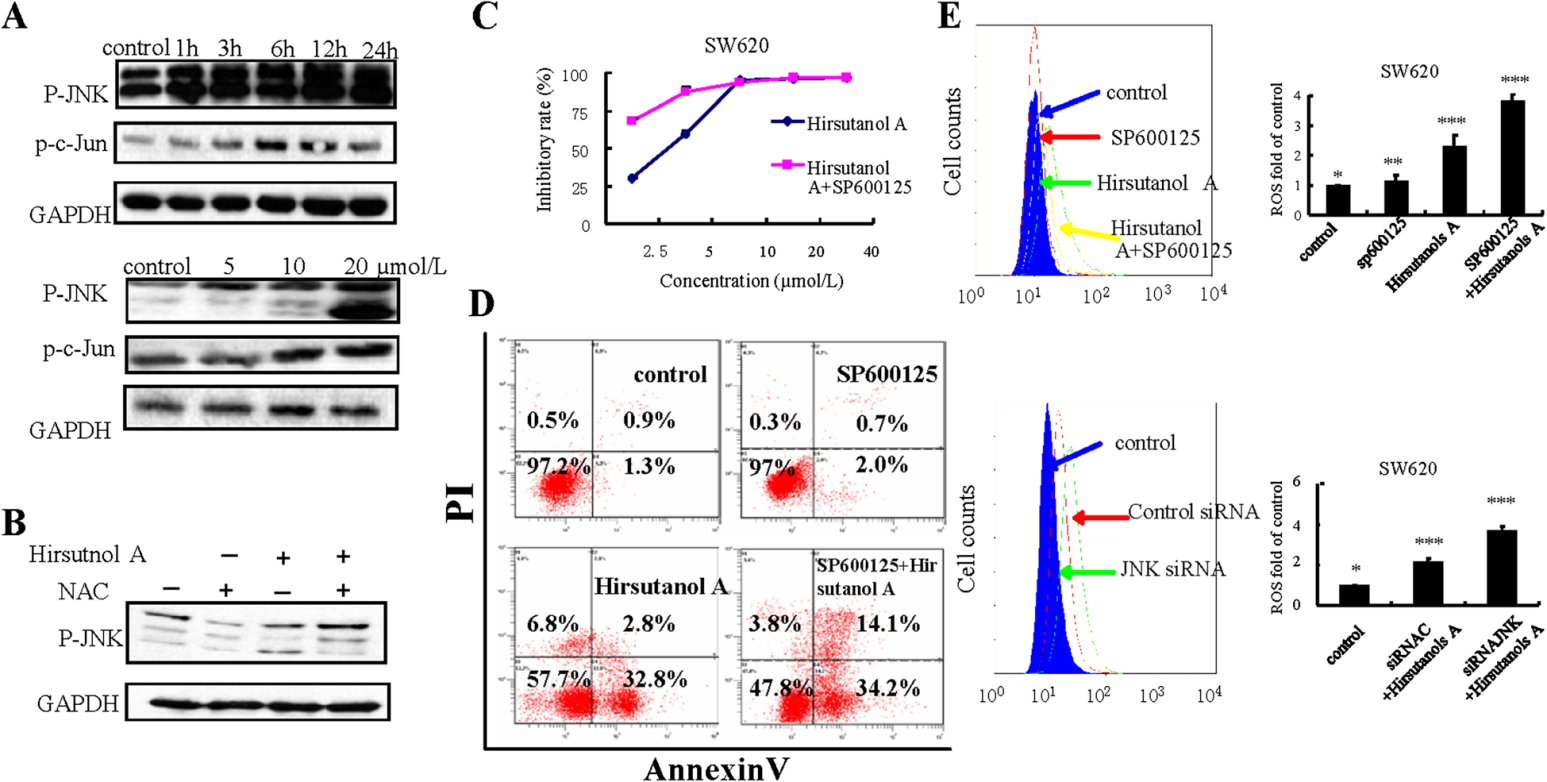
**Hirsutanol A activated JNK signaling pathway through elevating of ROS production and blockage of JNK signal pathway increased apoptosis and elevated ROS level induced by hirsutanol A. **SW620 cells were treated with 20 μmol/L hirsutanol A for indicated times or various concentrations of hirsutanol A for 24 h. The expression of p-JNK and p-c-Jun were detected by immunoblotting assay (**A**). SW620 cells were pre-incubated with NAC for 1 h, then treated with 20 μmol/L hirsutanol A for 24 h. The expression of p-JNK was determined by immunoblotting assay (**B**). SW620 cells were pre-incubated with SP600125 for 1 h , then treated with 20 μmol/L hirsutanol A for 72 h. Cell growth inhibition were detected by MTT assay (**C**). SW620 cells were pre-incubated with SP600125 for 1 h , then treated with 20 μmol/L hirsutanol A for 48 h. Cell apoptosis was detected by AnnexinV/PI analysis (**D**). SW620 cells were pre-incubated with SP600125 for 1 h or transfected with JNK- siRNA to block JNK signaling pathway, then treated with 20 μmol/L hirsutanol A for 3 h. Cellular ROS level was monitored by flow cytometry. Results are presented as means ± SD from 3 independent experiments. (** < 0.05 versus *; *** < 0.01 versus *) (**E**).

It has been reported that ROS can modulate several signaling pathways including JNK, Akt, NF-κB etc. [32,33]. Therefore, we explored the effect of increased ROS by hirsutanol A on JNK signaling pathway. JNK and c-Jun phosphorylation were significantly elevated in SW620 cells after treatment with hirsutanol A for 24 h (Figure 6A). However, this activation of JNK could be blocked by antioxidant agent NAC (Figure 6B). These suggested that JNK may be a downstream target of excessive ROS. In order to further explore the contribution of JNK signaling pathway to hirsutanol A-induced ROS accumulation, JNK signaling pathway was blocked using the small molecule JNK inhibitor SP600125 [34]. The percentage of AnnexinV- positive cells was 35.6% when cells were treated with hirsutanol A only, , whereas in parallel treatment in combination with SP600125, the percentage of AnnexinV-positive cells was 48.3%, suggesting that blocking of JNK signaling pathway promoted hirsutanol A-induced apoptosis (Figure 6C). The results also revealed that inhibiting JNK signaling pathway enhanced the growth inhibition effect induced by hirsutanol A (Figure 6D).

We further investigated the effect of activation of JNK signaling pathway on cellular ROS levels. Cellular ROS levels were remarkably increased in SW620 cells by JNK inhibitor SP600125 or JNK-siRNA (Figure 6E).These results suggested that activation of JNK could be one response to oxidant stress which protects cells from death via regulation of ROS in a negative feedback manner. It was not a classic mechanism involved in apoptosis.

### In vivo antitumor effect of hirsutanol A on human colon cancer cell SW620 xenografts

**Figure 7:**
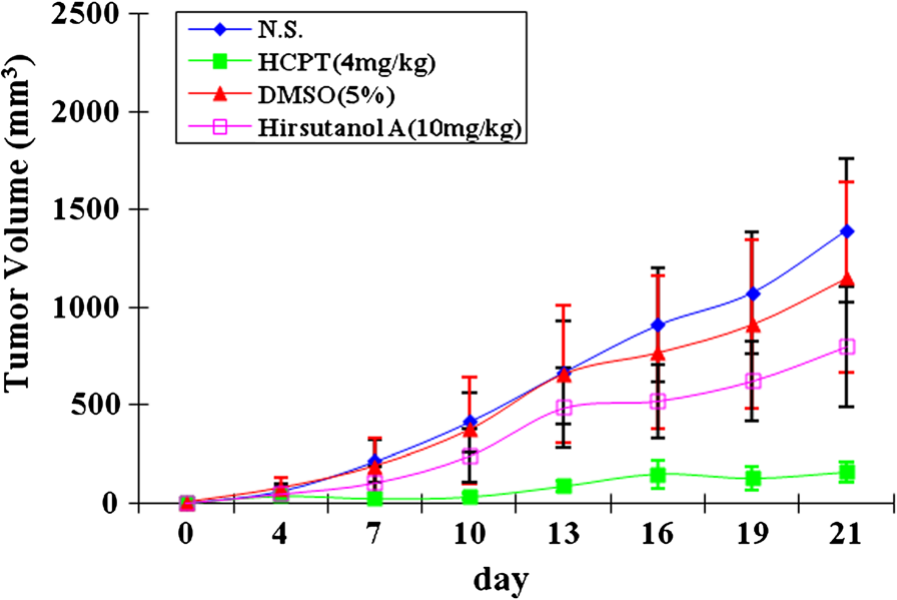
**In vivo anti**-**tumor effect of hirsutanol A on human colon cancer cells SW620 xenografts. **Cells were injected subcutaneously in BALB/C nude mice. After 6 days of implantment, BALB/C nude mice were treated with 5% DMSO, NS, hirsutanol A (10 mg/kg) or HPTC (4 mg/kg) for four weeks. Tumor sizes were measured twice weekly until the termination of the experiment (**A**). Mice were sacrificed on day 21.

To detect the antitumor activity of hirsutanol A in vivo, human colon cancer SW620 xenografts were established. The results showed that hirsutanol A at 10 mg/kg /d potently inhibited tumor growth (Figure 7).

## Discussion

Hirsutanol A is a novel sesquiterpene compound purified from fungus *Chondrostereum* sp. in *Sarcophyton tortuosum*. Our previous studies had demonstrated that hirsutanol A exhibited potent cytotoxic effect on some human cancer cell lines. In addition, we also confirmed that hirsutanol A could induce autophagical cell death by causing accumulation of ROS level in human hepatocellular carcinoma cells [18]. ROS inducer as an anticancer drug has received a lot of attention due to its selective effect on cancer cells but sparing normal cells [35]. To date, there are some ROS inducers targeting ROS generating system or ROS scavenging system [7,36]. However, most of them cannot enter clinical trials because of the high toxicity or poor bioavailability. Here, we reported that hirsutanol A could significant induce cell growth inhibition and apoptosis, elevate the level of ROS in both SW620 and MDA-MB-231 cells and suppress tumor growth in SW620 xenografts (Figures 2, 3 and 7). Some evidences supported that ROS as a potent oxidant agent could damage mitochondrial membrane to result in mitochondrial membrane potential disorder and release of cytochrome *c* from mitochondria which could further activate caspase-3, leading to mitochondria/cytochrome *c* –mediated apoptosis [37]. We had examined the mitochondrial membrane potential and the expression of cytochrome *c* in mitochondria and cytosol. The results showed that hirsutanol A could trigger the dysfunction of mitochondrial membrane potential and release of cytochrome *c* from mitochondria (Figure 5). Furthermore, we evaluated whether hirsutanol A-induced growth inhibition and apoptosis were evoked by accumulation of ROS. After treatment with NAC, a potent antioxidant agent that could prevent hirsutanol A-induced ROS accumulation [38], we found that cell growth inhibition and apoptosis remarkably decreased (Figure 4). As our data has clearly demonstrated that hirsutanol A could elevate intrinsic ROS level, and activate mitochondria/cytochrome *c* signaliing pathway to trigger apoptosis, further studies are required to elucidate if the release of cytochrome *c* is due to the elevated ROS induced by hirsutanol A.

ROS, which serves as a second messenger, can modulate several signaling pathways including JNK, Akt, NF-κB etc. [32,33]. In this study, we showed that hirsutanol A enhanced the phosphorylation levels of JNK and c-Jun dose-and time-dependently in SW620 cells (Figure 6A). Moreover, prevention of hirsutanol A-induced ROS accumulation by NAC could reverse the phosphorylation of JNK and c-Jun (Figure 6B). These data indicated that hirsutanol A-induced production of ROS activated JNK signaling pathway. JNK signaling pathway is involved in both stress-induced and chemotherapeutical drugs-induced apoptosis. However, inhibition of JNK signaling pathway by a special inhibitor SP600125 promoted the hirsutanol A-induced cell growth inhibition and apoptosis. Mass evidences verified that JNK signaling pathway is responsible for regulation of ROS level by activating c-Jun, a transcription factor, which further regulates the transcription of some target genes involved in redox such as NOX and SOD, etc. [39,40]. In our studies, we found that blockade of JNK signaling pathway by SP600125 and siRNA-JNK could significantly enhance hirsutanol A-induced ROS production, suggesting that hirsutanol A-induced activation of JNK signaling pathway regulated ROS level in a negative feedback manner. These evidences point us in the direction that treatment with hirsutanol A in combination with inhibitor of JNK may produce synergistic effect.

## Conclusion

In summary, hirsutanol A is a ROS generating agent which exerts anticancer effect via up-regulation of ROS level and activation of mitochondria/cytochrome *c* signaling pathway. Moreover, hirsutanol A could activate JNK signaling pathway. Activation of JNK signaling pathway did not mediate apoptosis; however, it could regulate ROS level in a negative feedback fashion which protects cells against oxidant stress induced cell death. Our results revealed that hirsutanol A may be a promising lead compound in future anticancer treatments.

## Competing interests

The authors declare that they have no competing interests.

## Authors’ contributions

**FY** and **WC** carried out the molecular genetic studies, immunoassays and drafted the manuscript. **HZ** carried out the immunoassays. **RD**, **JT**, **KW** and **DL** participated in the design of the study and performed the statistical analysis. FY, **GF** conducted the in vivo study. **WL**, **HL** and **XZ** conceived of the study, and participated in its design and coordination and helped to draft the manuscript. All authors read and approved the final manuscript.
